# Acetaminophen reduces the protein levels of high affinity amino acid permeases and causes tryptophan depletion

**DOI:** 10.1007/s00726-018-2613-8

**Published:** 2018-07-05

**Authors:** Angelina Huseinovic, Stefan J. Dekker, Bob Boogaard, Nico. P. E. Vermeulen, Jan M. Kooter, J. Chris Vos

**Affiliations:** 1 AIMMS, Division of Molecular Toxicology, Department of Chemistry and Pharmaceutical Sciences, VU University Amsterdam, De Boelelaan 1083, 1081 HZ Amsterdam, The Netherlands; 2 AIMMS, Department of Molecular Cell Biology, Section Genetics, VU University Amsterdam, De Boelelaan 1083, 1081 HZ Amsterdam, The Netherlands

**Keywords:** Acetaminophen, Tryptophan, Tyrosine, Nutrient starvation, Amino acid permeases

## Abstract

In yeast, toxicity of acetaminophen (APAP), a frequently used analgesic and antipyretic drug, depends on ubiquitin-controlled processes. Previously, we showed a remarkable overlap in toxicity profiles between APAP and tyrosine, and a similarity with drugs like rapamycin and quinine, which induce degradation of the amino acid permease Tat2. Therefore, we investigated in yeast whether APAP reduced the expression levels of amino acid permeases. The protein levels of Tat2, Tat1, Mup1 and Hip1 were reduced, while the expression of the general permease Gap1 was increased, consistent with a nutrient starvation response. Overexpression of Tat1 and Tat2, but not Mup1, Hip1 and Gap1 conferred resistance to APAP. A tryptophan auxotrophic strain *trp1Δ* was more sensitive to APAP than wild-type and addition of tryptophan completely restored the growth restriction of *trp1∆* upon APAP exposure, while tyrosine had an additive effect on APAP toxicity. Furthermore, intracellular aromatic amino acid concentrations were reduced upon APAP exposure. This effect was less prominent in ubiquitin-deficient yeast strains that were APAP resistant and showed a reduced degradation of high affinity amino acid permeases. APAP-induced changes in intracellular amino acid concentrations were also detected in hepatoma HepG2 cells indicating significance for humans.

**Electronic supplementary material:**

The online version of this article (10.1007/s00726-018-2613-8) contains supplementary material, which is available to authorized users.

## Introduction

Acetaminophen (*N*-acetyl-*p*-aminophenol, paracetamol, APAP), a widely used analgesic and antipyretic, is considered safe at therapeutic doses. However, an overdose induces hepatotoxicity and is one of the major causes of acute liver failure in the USA and Western Europe (Bernal et al. 2010). APAP can also cause toxicity at therapeutic doses as seen in Stevens–Johnsons syndrome and toxic epidermal necrolysis (Khawaja et al. 2012; Kim et al. 2014). Several studies reported a link between APAP use during pregnancy and the incidence of attention deficit hyperactivity disorder and hyperkinetic disorder in children (Liew et al. 2014), and long-term use was associated with increased incidence of cancer (Walter et al. 2011b, b) and asthma (Henderson and Shaheen 2013). Risk factors for APAP toxicity are concurrent medications, poor nutritional status, chronic alcohol abuse, obesity and non-alcoholic fatty liver disease (Larson et al. 2005; Michaut et al. 2014).

The major cause of APAP-induced hepatotoxicity and acute liver failure is due to the formation of the reactive metabolite *N*-acetyl-*p*-benzoquinone imine (NAPQI) by cytochrome P450, which binds to glutathione causing glutathione depletion and liver damage (Bessems and Vermeulen 2001; James et al. 2003). However, APAP can also be toxic without formation of NAPQI, as has been shown in mammalian cells (Jensen et al. 1996) and yeast (Srikanth et al. 2005). Furthermore, toxicity was also observed before glutathione depletion occurs (Shuhendler et al. 2014; Miettinen and Björklund 2014).

Previously, we used yeast *Saccharomyces cerevisiae* as a eukaryotic model organism to get more insight into NAPQI independent APAP toxicity, because yeast lacks the genes coding for drug-metabolizing P450 enzymes and is incapable of APAP metabolism and formation of NAPQI (Srikanth et al. 2005). Our study revealed that APAP toxicity depends on the cellular concentration of ubiquitin: ubiquitin depletion confers resistance to APAP, whereas ubiquitin overexpression caused sensitivity (Huseinovic et al. 2017b). Based on the correlation between ubiquitin levels and APAP-induced toxicity, we also performed a deubiquitinase (DUB) gene deletion screen (Huseinovic et al. 2017a). DUBs are enzymes that can reverse the process of ubiquitination, that often regulate ubiquitin levels and are involved in regulation of many essential cellular pathways such as DNA damage repair, internalization of membrane proteins, cell division and stress (Finley et al. 2012). The DUB screen showed that the APAP sensitivity and resistance growth phenotypes of the DUB deletion strains resembled those caused by other drugs, such as quinine (Khozoie et al. 2009), rapamycin (Beck et al. 1999), FTY720 (Welsch et al. 2003), FK506 (Schmidt et al. 1994) and ibuprofen (He et al. 2014). The latter drugs also induce tryptophan starvation by the ubiquitin-dependent degradation of the Tat2 amino acid permease (AAP).

Uptake of extracellular amino acids is regulated by high and low affinity AAPs (Regenberg et al. 1999; Ljungdahl and Daignan-Fornier 2012), whose expression in yeast is regulated by the amino acid sensing SPS and TOR pathways (Ljungdahl 2009; Shin et al. 2009). Membrane protein levels are regulated by the ubiquitin ligase Rsp5 followed by internalization from the plasma membrane and vacuolar degradation (Nikko and Pelham 2009). The internalization can be induced by a variety of environmental conditions, such as nutrient starvation (Khozoie et al. 2009), nutrient excess (Nikko and Pelham 2009), inhibition of the TOR pathway (Beck et al. 1999) or exposure to high pressure (Miura and Abe 2004). A variety of drugs have been identified that induce degradation of the high affinity permease Tat2, selective for aromatic amino acids. For example, quinine blocks tryptophan uptake through competitive inhibition, as it is structurally similar to tryptophan, and rapamycin causes tryptophan starvation indirectly by inhibiting the TOR pathway. During nutrient starvation, all high affinity AAPs are degraded, including Tat1 and Hip1, while the general AAP Gap1 is upregulated (Beck et al. 1999), regardless of the initial cause of the starvation response.

Since APAP treatment might result in aberrant amino acid sensing similar to an excess of tyrosine (Huseinovic et al. 2017a), we studied whether APAP can induce degradation of high affinity AAPs and increase the expression of general AAP Gap1, reminiscent of a nutrient starvation response. Also, we investigated the APAP-induced changes in intracellular amino acid concentrations.

## Materials and methods

### Yeast strains and media

Haploid deletion strains of *S. cerevisiae* with a BY4741 background (*MATa; ura3Δ0; leu2Δ0; his3Δ1; met15Δ0; gene∆::kanMX4*) and BY4733 (*MATa; ura3∆0; leu2∆0; his3∆200; met15∆0; trp1∆63; gene∆::kanMX4*) were obtained from EUROSCARF (Frankfurt, Germany). YPD medium contained 1% yeast extract, 2% peptone and 2% glucose. Selective YNB-URA medium contained 0.67% yeast nitrogen base, 2% glucose, 2% agar, 20 mg/l adenine (Ade), 20 mg/l uracil (Ura) and amino acids: 20 mg/l arginine (Arg), 20 mg/l histidine (His), 60 mg/l leucine (Leu), 30 mg/l lysine (Lys), 20 mg/l methionine (Met), 50 mg/l phenylalanine (Phe), 200 mg/l threonine (Thr), 20 mg/l tryptophan (Trp), and 30 mg/l tyrosine (Tyr). Plates contained additionally 2% agar. Yeast extract and peptone were obtained from Melford Laboratories Ltd. (Ipswich, UK). Amino acids, yeast nitrogen base, agar and glucose were purchased from Sigma-Aldrich, St. Louis, USA.

### Plasmids and transformations

PCR amplifications of genes *TAT1*, *MUP1*, *HIP1* and *GAP1* were made using genomic DNA of BY4741 strains as template. The genes with an N-terminal HA-tag were cloned in two steps. First, PCR amplification of the gene coding region, without start codon and containing stop codon plus ~ 300 bp downstream sequence, was cloned into a Yeplac195 based plasmid (2*µ*, *URA3*) containing a 3 × HA tag (kindly provided by Jan Paul Bebelman). Subsequently, the gene-specific promoter, ~ 1000 bp sequence upstream of the coding region, was cloned upstream of the HA-tagged coding region. The clones were identified by restriction digestion and additionally sequenced to verify the correct DNA sequence. The primers sequences are available on request. The plasmids YEplac195 (2*µ*, *URA3*) containing HA-tagged versions of *TAT2* and *TAT2*^5*K* >* R*^ genes and YCpac33 (CEN, *URA3*) containing HA-tagged version of *TAT2* were a kind gift from Prof. M. Hall. The plasmids were transformed into yeast cells by the freeze–thaw method as previously described (Klebe et al. 1983).

### Western blotting

For the determination of HA-tagged protein levels from multi copy plasmids (2*µ*, *URA3)*, overnight cultures (16 h) were grown at 30 °C in YNB-URA medium to OD_600_ of 0.5 in 10 ml YNB for each sample. Prior to APAP exposure, the cultures were incubated at 37 °C for 3 h. Subsequently, the cells were treated with 0, 50, 75 and 100 mM APAP (Sigma-Aldrich, St. Louis, USA) for 1 h. The OD_600_ was measured and for each sample the volume of cells equivalent to OD_600_ of 10 was harvested by centrifugation and were frozen at − 20 °C. The cell pellets were dissolved in 200 µl Laemmli buffer without bromophenol blue (50 mM Tris–HCl pH 6.8, 2% SDS, 10% glycerol, 12.5 mM EDTA, supplemented with 1 mM PMSF, 10 mM DTT and protease inhibitor (EDTA-free Complete, Roche) and cells were mechanically broken by vortexing with acid washed beads (Sigma-Aldrich) for 6 min. Subsequently, the lysates were incubated at 37 °C for 10 min and the protein extract was separated from insoluble cell debris and beads by centrifugation. Aliquots were diluted 1:40 in water and the amount of total protein was determined by a protein assay (Bio-Rad). Subsequently, 0.02% bromophenol blue was added to the samples and a Western blot was performed. Briefly, equal amounts of protein were loaded on 10% bis–Tris gels and the proteins were separated in MES buffer (Life technologies).

For the determination of HA-Tat2 expression from a single copy plasmid (CEN, *URA3*), the cells were grown and treated with APAP as described above and P13 membrane fractions were isolated as described previously (Abe and Iida 2003). Briefly, the cells were washed with 10 mM NaN3/10 mM NaF and lysis buffer (50 mM Tris–HCl, 5 mMEDTA, 10 mM NaN3, 1 mM PMSF and protease inhibitor (EDTA-free Complete, Roche). After washing, the cells were resuspended in 200 µl lysis buffer and broken with glass beads (vortexed for 6 min) and centrifuged for 2 min at 300*g* to remove unbroken cells and debris. Then, 100 µl of the cleared lysates was mixed with an equal volume of STE20 buffer (20% sucrose, 50 mM Tris–HCl pH 7.5, 5 mM EDTA) and centrifugated at 13,000 g for 10 min to yield a P13 (pellet) fraction. P13 fractions were resuspended in 30 µl of adjusted 1 × Laemmli sample buffer (60 mM Tris–HCl pH 6.8, 5% SDS, 10% glycerol and 5% ß-mercaptoethanol) at 37 °C for 10 min. The protein concentration was measured by nanodrop before 0.004% bromophenol blue was added. Equal amounts of protein (~ 120 µg) were loaded on 12% Tris–HCl gels and the proteins were separated in Tris–Glycine-SDS buffer. The proteins were blotted on a 0.45 µm PVDF membrane (Thermo scientific). Protein loading was controlled by Ponceau S staining. The membrane was blocked in 5% skimmed milk (Sigma-Aldrich, St. Louis, USA) resolved in TBST (20 mM Tris, 150 mM NaCL, 0.1% Tween 20) and washed in TBST. HRP conjugated HA-antibody and anti-actin antibody used for detection were obtained from Santa Cruz (sc-8017) and Merck Millipore (MAB1501), respectively. The secondary antibodies rabbit anti-mouse HRP conjugated used for actin detection was ordered from Abcam (ab97046). The detection was performed by ECL reaction (Pierce) in a Bio-Rad imager.

### Spot dilution assay

For the AAPs overexpression experiments, WT cells were transformed with plasmids (2*µ*, *URA3*) of the HA-tagged genes of Tat2, Tat1, Hip1, Mup1 and Gap1 and grown overnight in YNB-URA medium at 30 °C. Subsequently, cultures were diluted to an OD_600_ of 0.05 and additional fivefold serial dilutions were made. The cells were plated on YPD plates with and without 70 mM APAP and incubated for 3 days at 37 °C. For the *trp1∆* sensitivity determination, WT (BY4741) and *trp1∆* (BY4733) cells were grown overnight in YPD medium at 30 °C and fivefold serial dilutions of cell suspensions with optical density OD_600_ of 0.05 were made and plated on YPD plates with or without 50 mM APAP or 200 ng/ml rapamycin (Sigma-Aldrich, St. Louis, USA) and incubated for 3 days at 37 °C. The cells were spotted on YPD agar plates using a 96-well replica plater (Sigma-Aldrich).

### Growth rate measurements

The yeast cells were grown at 30 °C in YPD medium to exponential growth phase, washed in water and resuspended in YNB medium with predefined amino acid concentrations (see the composition of YNB medium). The treatments were performed in duplicate in a 96-well plate in a final volume of 200 µl in medium containing 40 mM APAP and additional onefold of different amino acids. The cells were grown for 19 h at 37 °C and the OD_600_ was measured every 20 min. The controls were cells grown in medium with or without 40 mM APAP. The average growth curves and standard errors of the two duplicates were calculated.

### Yeast cells sample preparation for HPLC

WT, *ubi4∆, doa4∆ and doa1∆* yeast cells were grown in duplicate in 5 ml YPD medium overnight at 30 °C. Subsequently, the OD_600_ was measured and the cultures were diluted in fresh YPD medium to OD_600_ of 0.5 to a final volume of 20 ml and pre-incubated for 3 h at 37 °C to reach exponential phase. For the drug treatments, each culture was separated in two 10 ml cultures and, subsequently, 10 ml of pre-warmed YPD medium with or without 150 mM APAP was added to the final concentration of 0 and 75 mM APAP. The cells were incubated 37 °C; WT cells for 10, 30 and 60 min and *ubi4∆, doa4∆* and *doa1∆* for 60 min. Subsequently, the cultures were cooled on ice and washed twice in ice-cold milliQ water. After washing, the cells were resuspended in 1.1 ml ice-cold milliQ water, 1 ml was centrifuged at 2000*g* for 2 min and the pellets were frozen at − 20 °C. The remaining 100 µl was used to measure OD_600_ and this value was used to resuspend the frozen pellets in appropriate amount of milliQ water containing 100 µM of dl-2-aminobutyric acid (TCI, Tokyo, Japan) as internal standard to obtain a cell density of OD_600_ is 8 per 1 ml. Subsequently, the cell suspension was incubated at 90 °C for 15 min and centrifuged for 2 min. The supernatant containing free amino acids was collected and filtered through a small volume 0.2 µm filter (purchased by Phenomenex) and further prepared for HPLC analysis by diethyl ethoxymethylenemalonate (DEEMM) derivatization.

### HepG2 cells sample preparation for HPLC

HepG2 cells were cultured in Dulbecco’s Modified Eagle’s Medium (DMEM; Lonza, Basel, Switzerland) supplemented with 10% Fetal Bovine Serum (FBS) (Lonza), 1% penicillin/streptomycin (Lonza), 1% l-glutamine (Lonza) and 1% non-essential amino acids (Sigma-Aldrich). During routine passage, 80% confluent cells were washed with phosphate-buffered saline (PBS) solution (Lonza), trypsinized with Trypsin–EDTA (Lonza). Prior to APAP treatment, 1 × 10^6^ HepG2 cells/well in a 6-well plate were seeded and grown at 37 °C in a monolayer to the confluency of ~ 70%. Subsequently, the cells were washed with PBS and DMEM medium with 10% FBS containing 0, 10 and 20 mM APAP and incubated for 2 h. The medium was removed by aspiration and the cells were washed two times with warm PBS solution. Subsequently, 500 µl water containing 100 µM dl-2-aminobutyric acid (internal standard) was added in each well, the cells were harvested by scraping, incubated at 90 °C for 15 min, vortexed and centrifuged at high speed for 15 min. The supernatant containing released free intracellular amino acids was collected, filtered through a 0.2 µm filter and prepared for HPLC by DEEMM derivatization.

### Amino acid DEEMM derivatization and HPLC measurements

The amino acid derivatization was performed according to (Alaiz et al. 1992). Briefly, 400 µl of the amino acid extract obtained from yeast of HepG2 cells was mixed with 1 ml of 150 mM borate buffer pH 9.0 and 0.8 µl DEEMM (Sigma-Aldrich, St. Louis, USA) and subsequently incubated at 50 °C for 1 h by shaking at 1400 rpm. The samples were centrifuged for 15 min at 20,800*g*. 50 µl of the samples were injected by a Gilson 234 auto injector connected to two Gilson 305 pumps onto a 250 × 4.8 mm Zorbax column connected to a C18 guard column. The UV signal at 280 nm was detected with a photodiode array detector (SPD-20A Shimadzu). Eluent *A* was 10 mM acetate pH 5.8 in ddH_2_O and eluent *B* was 100% acetonitrile. The flow rate was set to 0.9 ml/min. The HPLC program was optimized to obtain an optimal amino acid peak separation by adjusting the eluent *B* percentage (%) at different time points (Table 1).

**Table 1:** HPLC program used for amino acids determination

Time point (min)	0	10	20	30	40	45	56	58	60	61	72
Eluent *B* (%)	1	5	9	13	18	19	30	50	60	95	1

### HPLC data analysis and statistics

The obtained HPLC chromatograms were analyzed with Shimadzu CLASS-VP Chromatography Data System 4.3 software (Kyoto, Japan), which quantified each area peak for each amino acid with and without APAP treatment and the values were exported in an excel file. The amino acid retention times were determined by HPLC analysis of each individual amino acid standards, which were derivatized the same way as the samples. The peak area values were normalized against the values of the internal standard dl-2-aminobuttylic acid. The relative increase or decrease for each amino acid with APAP treatment was calculated from the average of the duplicate measurements according to the formula $R = \frac{{\left( {X - Y} \right)}}{Y} = \frac{X}{Y} - 1 [\% ]$. The standard error was calculated according to the general rule for error calculation of multiplication or division of measured quantities with formula $\delta R = \left| R \right| \cdot \sqrt {\left( {\frac{\delta X}{X}} \right)^{2} + \left( {\frac{\delta Y}{Y}} \right)^{2} }$, where *X* and *Y* are the average values with and without APAP treatment, respectively, *δX* and *δY* are their corresponding standard deviations, and *R* is the relative increase or decrease percentage when compared to the non-treated cells.

## Results

### APAP induces degradation of Tat2, Tat1, Mup1 and Hip1, and upregulates Gap1

**Fig. 1:**
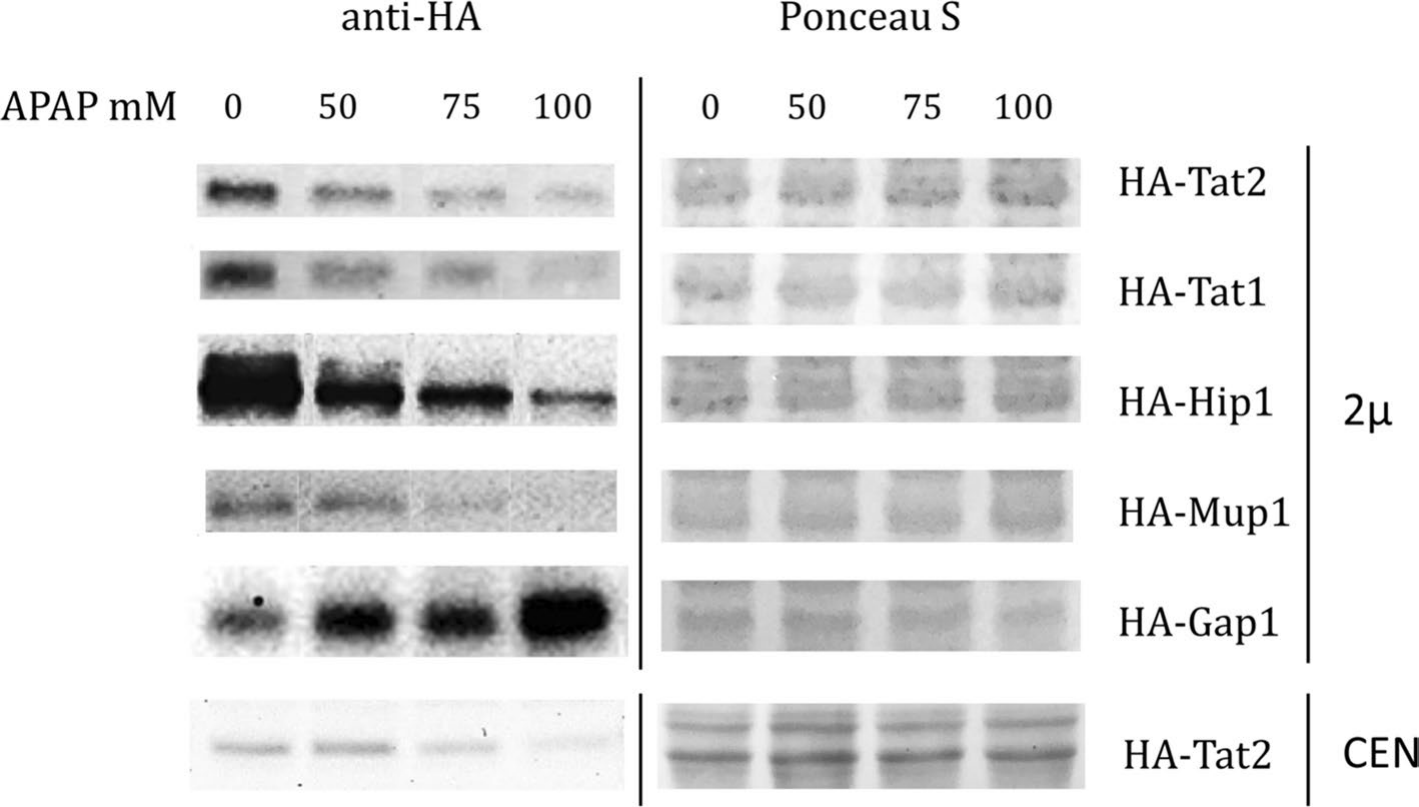
APAP induced concentration-dependent degradation of Tat2, Tat1, Hip1 and Mup1, while Gap1 expression was increased. The WT cells containing a multi copy plasmid (2*µ*, *URA3*) or a single copy plasmid (CEN, *URA3*) with corresponding HA-tagged genes were grown exponentially in YNB-URA and treated with 0, 50, 75 and 100 mM APAP for 1 h. Equal amounts of protein were loaded. The blots were assayed for HA-Tat2, HA-Tat1, HA-Hip1, HA-Mup1 and HA-Gap1 expression (on the left) and the Ponceau *S* staining was used as the loading control (on the right)

In the previously performed DUB toxicity screen (Huseinovic et al. 2017a), we showed similarities between the DUB toxicity profiles of tyrosine, APAP and drugs causing degradation of Tat2, such as quinine (Khozoie et al. 2009) and ibuprofen (He et al. 2014). To determine whether APAP can also induce degradation of amino acid permeases, we tested the expression levels of several high affinity AAPs during APAP treatment at concentrations that reduce the growth rate. We used for this WT cells transformed with plasmids (2*µ*, *URA3*) expressing HA-tagged versions of Tat2 (Trp, Tyr and Phe permease), Tat1 (Trp, Tyr, Leu, Val and Thr permease), Hip1 (His permease), Mup1 (Met permease) and of the general AAP Gap1. The cells were grown for 1 h in YNB-URA medium containing 0, 50, 75 and 100 mM of APAP and protein levels were determined by Western blotting. The results showed that the abundance of Tat2, Tat1, Hip1 and Mup1 declined in a dose-dependent manner upon APAP exposure (Fig. 1). In contrast, Gap1 levels increased in a dose-dependent manner. A similar effect of Tat2 degradation upon APAP exposure is also shown in WT cells expressing HA-TAT2 from a single copy plasmid (CEN, *URA3*) (Fig. 1, bottom panel), indicating that the observed effect is not restricted to overexpression conditions. Concurrent high affinity AAPs degradation and induction of general AAP Gap1 suggest that APAP caused a nutrient starvation response.

### Tat2 and Tat1 overexpression confers resistance to APAP

**Fig. 2:**
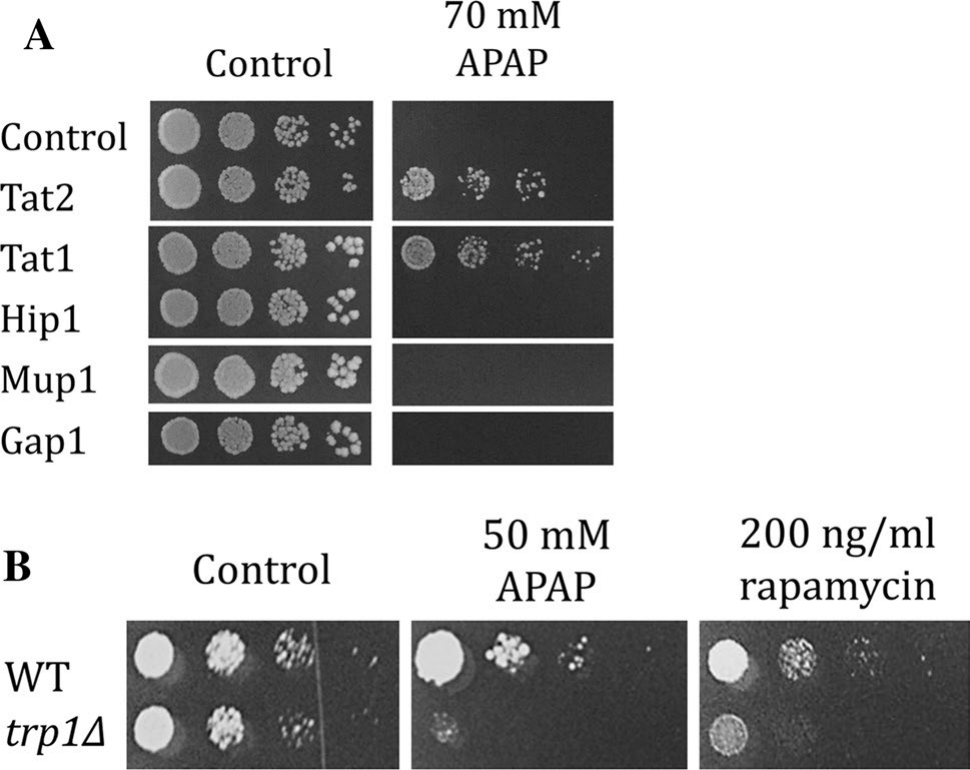
Tat2 and Tat1 overexpression confers APAP resistance and a *trp1∆* strain is hypersensitive. **a** WT cells were transformed with the plasmid (2*µ*, URA3) containing the HA-tagged gene of Tat2, Tat1, Hip1, Mup1 and Gap1. The cells were grown overnight in YNB-URA medium and fivefold serial dilutions of cell suspensions with optical density OD_600_ of 0.05 were plated on YPD plates containing 0 and 70 mM APAP and incubated for 3 days at 37 °C. **b** WT (BY4741) and *trp1∆* (BY4733) cells were grown overnight in YPD medium and fivefold serial dilutions of cell suspensions with optical density OD_600_ of 0.05 were plated on YPD plates containing 50 mM APAP and 200 ng/ml rapamycin. The plates were incubated for 3 days at 37 °C

APAP induced degradation of high affinity AAPs Tat1, Tat2, Hip1, Mup1, which was likely to limit amino acid availability in the cell. Therefore, we wanted to see whether the overexpression of these permeases could counteract the APAP-induced growth reduction. For that purpose, we expressed the permeases in WT cells from a multicopy plasmid using a HA N-terminal tag, which has been used successfully before (Beck et al. 1999). We tested these cells in a spot dilution assay with and without APAP. Interestingly, only Tat1 and Tat2 overexpression, which are both involved in uptake of Trp and Tyr, conferred APAP resistance, while overexpression of Hip1 (His uptake), Mup1 (Met uptake) and Gap1 (general amino acid permease) did not rescue the cell growth upon APAP exposure (Fig. 2a). In our previous research (Huseinovic et al. 2017a) we already showed that addition of extra Trp provided growth rescue upon APAP exposure. Therefore, we wanted to see whether a Trp auxotrophic strain would be sensitive to APAP. Indeed, the Trp auxotrophic strain BY4733 was hypersensitive to APAP when compared to its *TRP1* counterpart BY4741 (WT). Rapamycin caused a similar sensitivity and has been shown to induce Trp starvation and Tat1 and Tat2 degradation through the inhibition of TOR pathway (18) (Fig. 2b).

### Tat2 expression level is stabilized in APAP resistant strains *ubi4∆*, *ubp6∆* and *doa1∆*

**Fig. 3:**
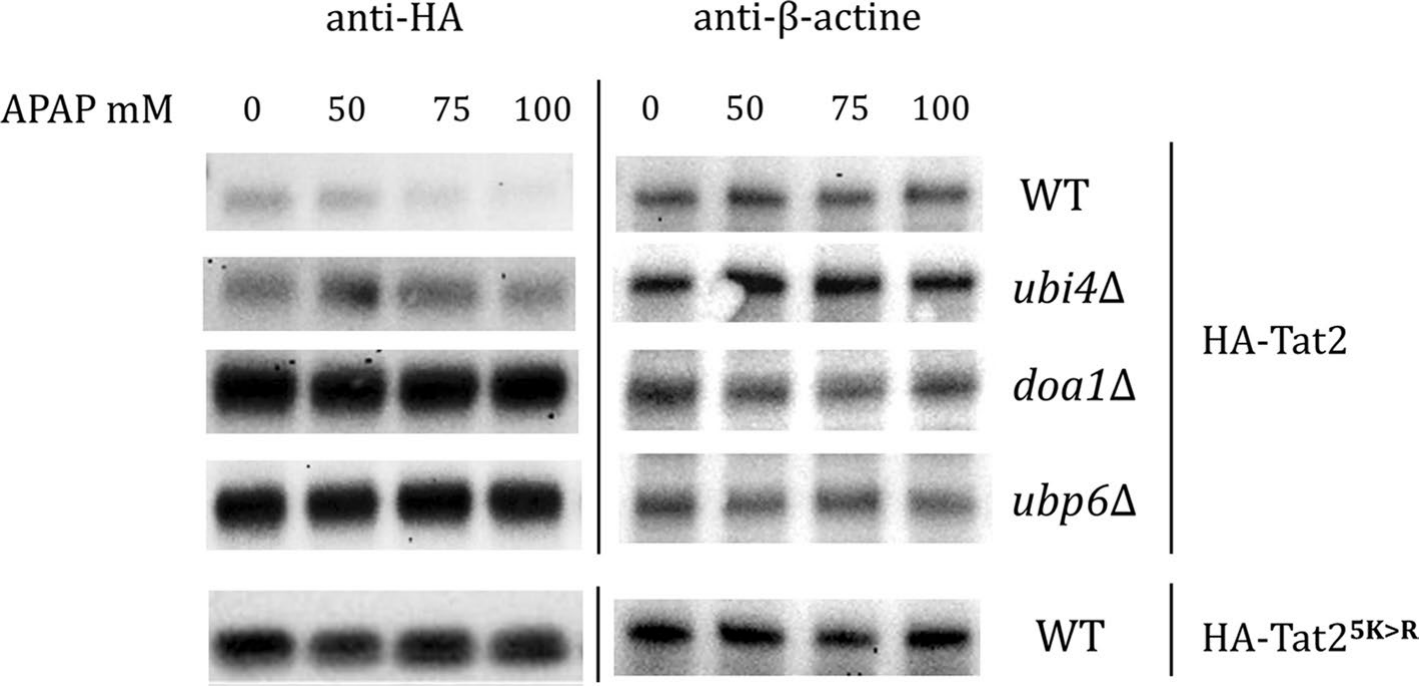
APAP induces degradation of Tat2. APAP treatment induced degradation of Tat2 in WT cells, while the expression level of Tat2 was unaffected in ubiquitin-deficient strains *ubi4∆, doa1∆* and *ubp6∆*. The expression level of *TAT2*^5*K *> *R*^ mutant was also constant in WT cells. The cells containing multi copy plasmid (2*µ*, *URA3*) with *HA*-*TAT2* gene (all strains) or *HA*-*TAT2*^5*K *> *R*^ gene (WT) were grown exponentially in YNB-URA medium and treated with 0, 50, 75 and 100 mM APAP for 1 h. Total protein levels were determined by protein assay (Bio-Rad) and equal amounts of protein were loaded. The blots were assayed for HA-Tat2 expression (on the left). ß-actine expression was used as the loading control (on the right)

In our previous research (Huseinovic et al. 2017a, b), we showed that APAP toxicity depends on cellular ubiquitin levels and that ubiquitin-deficient strains, such as *ubi4∆*, *doa1∆, doa4∆*, *ubp6∆* and *∆ubp14,* conferred resistance to APAP (Huseinovic et al. 2017b). Since it is known that the Tat2 expression level is stabilized in deletion strains *doa4∆*, *ubp6∆* and *ubp14∆* under high pressure (Miura and Abe 2004), and based on our current finding that only Tat1 or Tat2 overexpression conferred resistance to APAP, we hypothesized that the Tat2 expression level is stabilized in the APAP resistant mutants during APAP treatment. To determine the levels of Tat2 upon APAP treatment, we expressed a HA-tagged *TAT2* in WT, *ubi4∆*, *ubp6∆* and *doa1∆* cells and determined its abundance after 1 h exposure to APAP. We also tested the expression of HA-Tat2^5*K* > *R*^ mutant in WT cells, which lacks five lysines and cannot be ubiquitinated (Beck et al. 1999). The role protein ubiquitination by E3 ubiquitin ligase Rsp5 in protein degradation of AAPs (i.e. Mup1, Tat1 and Tat2) has been well-established (Nikko and Pelham 2009; Suzuki et al. 2013). Indeed, the Western blot results revealed that the levels of Tat2 were unaffected and higher in *ubi4∆*, *doa1∆* and *ubp6∆* strains when compared to the WT, probably due to the reduced levels of ubiquitin and/or reduced vacuolar degradation of transmembrane proteins (*doa1∆*). Similarly, the amount of HA-Tat2^5*K* > *R*^ mutant was also constant upon APAP exposure, demonstrating the role of ubiquitination in Tat2 degradation. The apparent higher levels of Tat2 expression in the deletion strains under normal conditions may also be due to the reduced ubiquitin levels in the cell (Fig. 3).

### The effects of individual amino acids on APAP-induced growth inhibition

**Fig. 4:**
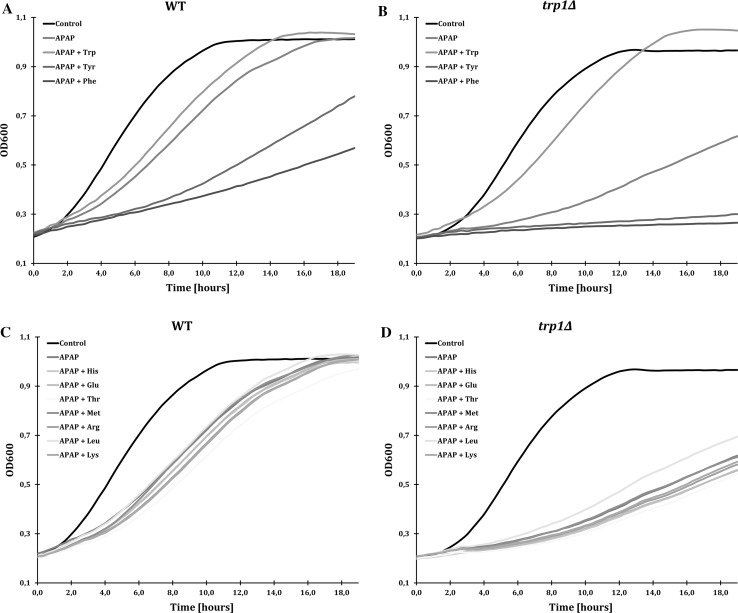
Effect of amino acids supplements on APAP-induced growth inhibition. The WT and *trp1Δ* cells were grown in a 96-well in YNB medium and treated with 40 mM APAP with and without onefold addition of single amino acids: **a** and **b** Trp, Tyr and Phe, and **c** and **d** His, Glu, Thr, Met, Arg, Leu and Lys. OD_600_ measurements were performed every 20 min for 19 h. The standard errors from two individual measurements are not presented, but were not higher than 5% (see Online Resource 1)

Because Tat1 and Tat2 overexpression conferred APAP resistance and a *trp1∆*-strain was more sensitive to APAP, we investigated the effect of addition of individual amino acids on growth of WT and *trp1∆* cells during APAP exposure. We tested the growth effect of addition of a surplus of the aromatic amino acids Trp and Tyr (transported by Tat1 and Tat2) and Phe (transported by Tat2). We also tested addition of His, Met or Leu, since WT and *trp1∆* cells are auxotrophic for these amino acids, and their transporters (Hip1, Mup1 and Tat1, respectively) were downregulated during APAP exposure (Fig. 1). For comparison and as a control, we also tested the growth effects of Arg and Lys (transported by Lyp1), Glu (transported by Gap1) and Thr (transported by Agp1, Gnp1 and Tat1). The cells were grown in 96-well plates in liquid YNB medium containing APAP with or without onefold extra addition of each amino acid based on the concentrations that are normally present in YNB medium (see Materials and methods) and the cell growth was measured as an increase in optical density (OD_600_) (Fig. 4). Note that onefold extra addition of each amino acid without APAP did not have a growth effect on the cells (data not shown). Both WT and *trp1∆* strains exhibited impaired growth in the presence of APAP, but the effect on *trp1∆* cells was much stronger, similar to what was already observed on a solid culture (Fig. 3b). Addition of Trp enhanced growth, especially in the *trp1∆* cells. In contrast, addition of Tyr and Phe increased the APAP-induced toxicity and almost completely inhibited growth (Fig. 4a, b).

Addition of Arg, Lys, Glu and Thr did not have a major effect on growth rate (Fig. 4c, d). In conclusion, these experiments showed that only addition of Trp was sufficient to rescue growth of *trp1∆* upon APAP exposure, while addition of Tyr and Phe increased APAP toxicity leading to an almost complete growth arrest. These results suggest that APAP causes a growth-limiting shortage of intracellular Trp. The raw data and the calculations are presented in Online Resource 1.

### The role of Tat1 and Tat2 permeases in APAP sensitivity

**Fig. 5:**
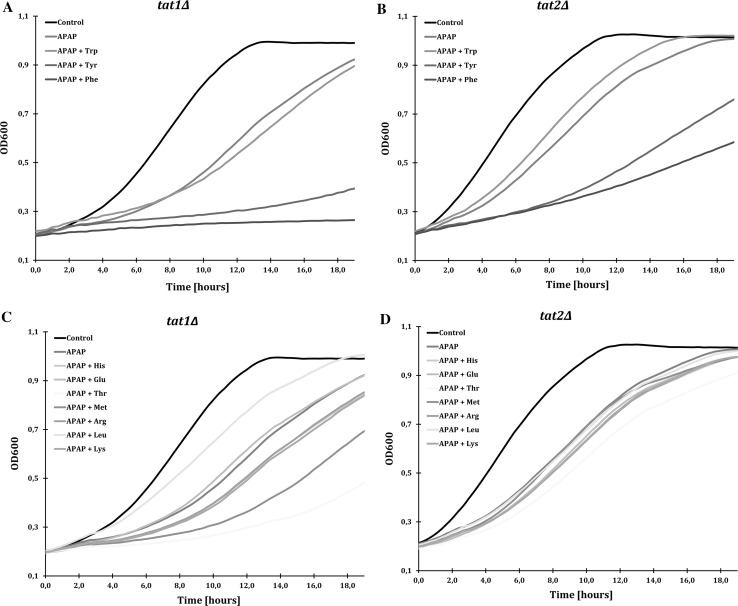
*TAT1* is required for growth rescue by tryptophan upon APAP exposure. The *tat1Δ tat2Δ* cells were grown in a 96-well plate in YNB medium and treated with 40 mM APAP with and without the addition of onefold of single amino acids: **a** and **b** Trp, Tyr and Phe, and **c** and **d** His, Glu, Thr, Met, Arg, Leu and Lys. The OD_600_ measurements were performed each 20 min for 19 h. The standard errors from two individual measurements are not presented, but were not higher than 5% (see Online Resource 1)

**Fig. 6:**
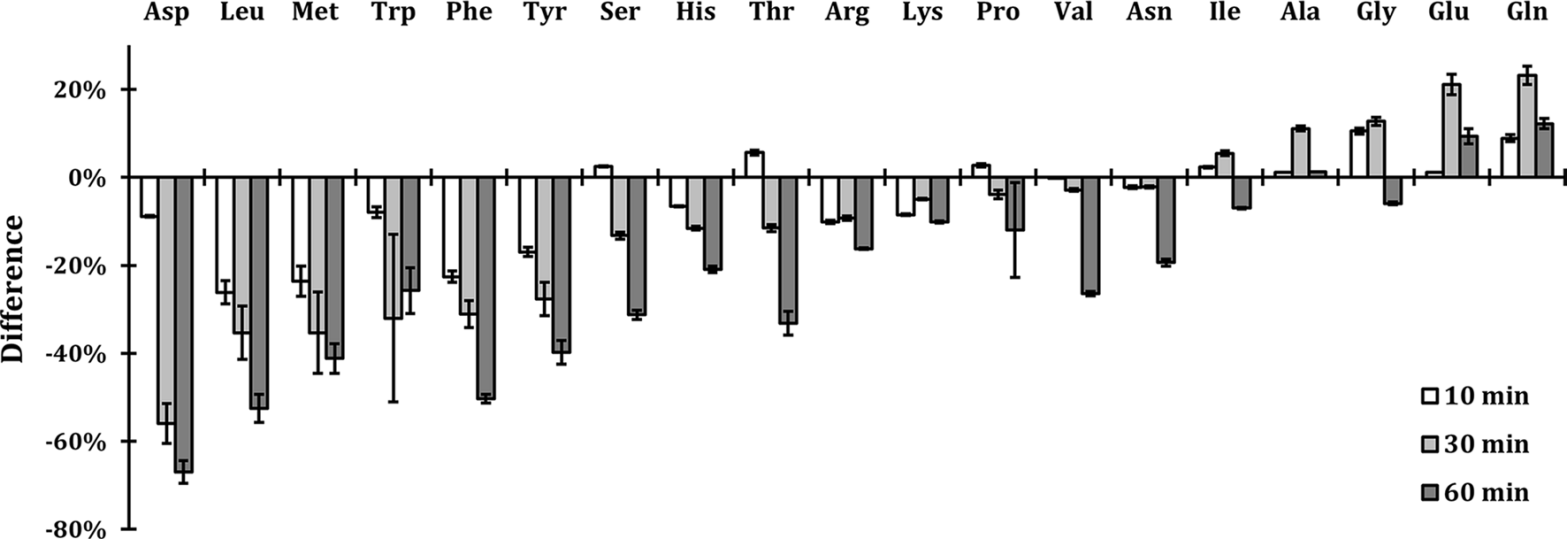
APAP-induced changes in amino acid levels. WT cells were grown exponentially in YPD medium and treated with 75 mM APAP for 10, 30 and 60 min at 37 °C. The intracellular amino acid levels were measured by HPLC. The bars represent relative increase decrease in intracellular amino acid levels compared to the non-treated cells. The error bars present standard error of two individual measurements. The amino acids were ordered based on the highest decrease at the 30 min time point

Next, we investigated the relative importance of *TAT1* and *TAT2*. Therefore, we tested the growth effect of addition of Trp, Tyr or Phe on *tat1∆* and *tat2∆* strains during APAP exposure. Notably, *tat2∆* showed similar growth as the WT, with a slight growth rescue upon addition of Trp, and enhanced toxicity after Tyr or Phe addition (Fig. 5b, d). However, *tat1∆* showed higher sensitivity to APAP, almost complete growth impairment after addition of Tyr and Phe, but no growth rescue after Trp addition (Fig. 5a) indicating that Tat1 is the main transporter of Trp into the cells under these conditions. Addition of Glu, Lys and, especially, Met or Thr had a negative growth effect upon APAP exposure in *tat1∆* (Fig. 6c), suggesting a complex role of Tat1 in maintaining amino acid availability. The raw data and the calculations are presented in Online resource 1.

### Changes in intracellular amino acid levels upon APAP treatment

Our findings that APAP induced the degradation of high affinity permeases (Fig. 1) and that APAP toxicity can be rescued by addition of Trp (Fig. 4) prompted us to determine the effect of APAP on intracellular levels of all amino acids. For that purpose, we cultured WT cells in liquid YPD culture in the presence or absence of APAP and harvested after 10, 30 and 60 min. The level of each amino acid upon APAP treatment was determined by HPLC analysis relative to the non-treated cells. The results showed that already after 10 min of APAP treatment there was a decrease in the intracellular levels of Leu, Met, Phe, Tyr, Arg, Asp, Lys, Trp, and His, while Gly, Gln and Thr showed increased levels (Fig. 6). After 30 min of APAP treatment, an even higher decrease of most amino acid levels was measured, except for Ile, Ala, Gly, Glu and Gln, while after 60 min of exposure all amino acid levels were decreased except for Glu, and Gln, which were increased (Fig. 6). Note that we did not present Cys, due to the low levels of detection. It is known, that Cys is a low abundant amino acid in yeast (Romagnoli et al. 2015). The complete set of raw data and calculations are presented in Online resource 2.

### Differences in amino acid levels between WT and APAP resistant strains

**Fig. 7:**
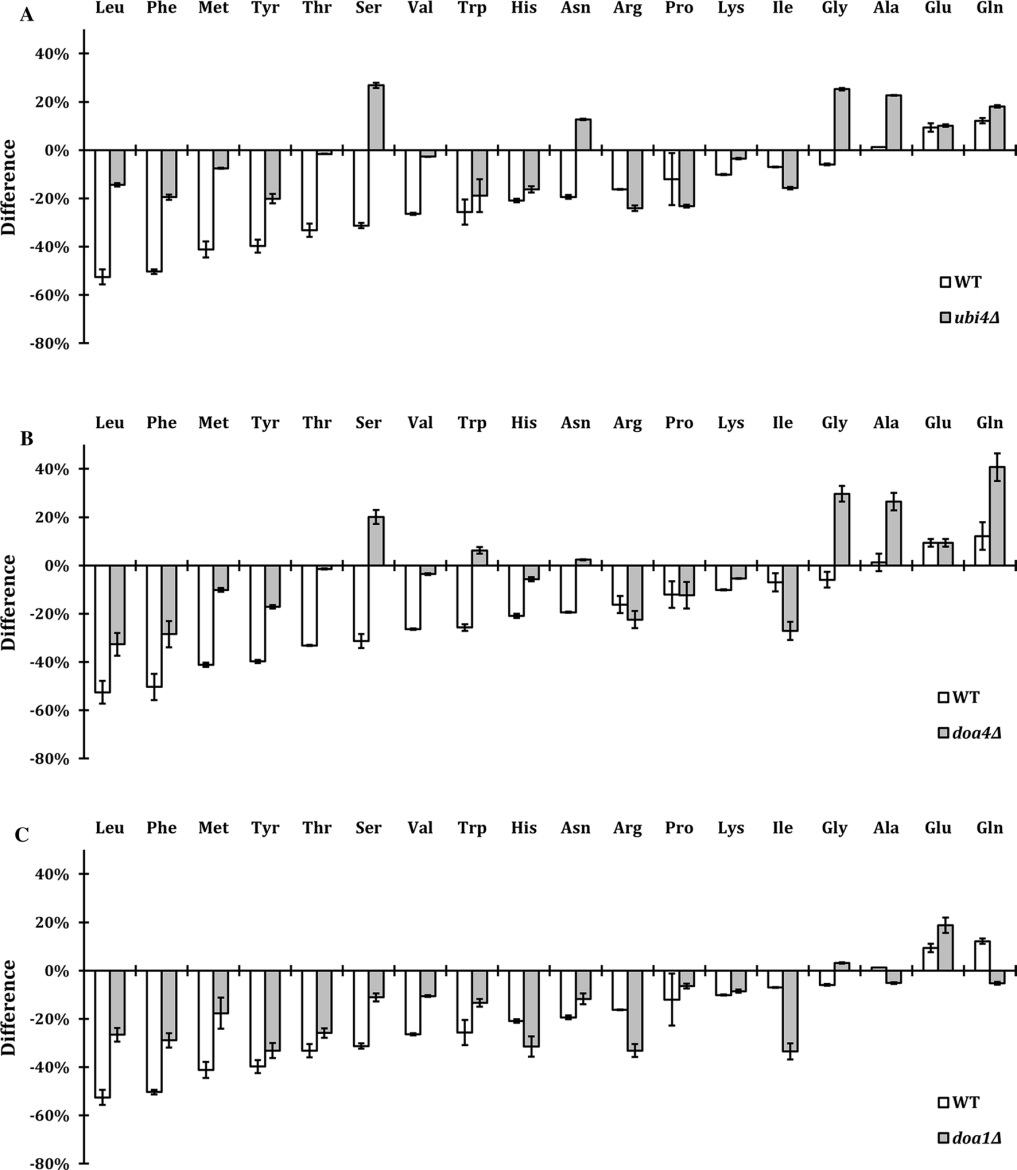
Relative decrease in amino acid levels upon APAP exposure compared to the wild-type cells is lower in ubiquitin-deficient strains *ubi4∆, doa4∆* and *doa1∆*. The cells were treated with 75 mM APAP for 1 h at 37 °C. The values are relative increase/decrease of intracellular levels compared to the non-treated cells. The gray bars represent *ubi4∆* **a**, *doa4∆* **b** and *doa1∆* **c**, and white bars represent WT. The error bars represent the standard error of two independent measurements. The amino acids were ordered based on the highest amino acid level decrease in the WT strain

Since the expression levels of Tat2 were stable in ubiquitin-deficient strains (Fig. 2b) (Miura and Abe 2004), we determined the effect of APAP exposure on the amino acids levels in several of these deletion strains, i.e., *ubi4∆*, doa4∆ and doa1∆. The cells were grown exponentially, exposed to APAP for 1 h and the intracellular amino acid levels were measured by HPLC (Fig. 7). Most amino acid levels in the ubiquitin-deficient strains were less decreased than in wild-type cells, especially the amino acids Leu, Phe, Met, Tyr, Ser, Val, Trp and Asn. Strains *ubi4∆* and *doa4∆* showed similar profile of increase/decrease for all amino acid levels, indicating that the APAP resistance of *doa4∆*, similar to *ubi4∆*, is probably due to the ubiquitin deficiency. In contrast to WT, Gly levels were increased in *ubi4∆, doa1∆* and *doa4∆* upon APAP exposure. Ser and Ala were markedly increased in only *ubi4∆* and *doa4∆*. The complete set of raw data and calculations are presented in Online resource 3.

### Intracellular amino acid concentration in HepG2 cells upon APAP treatment

**Fig. 8:**
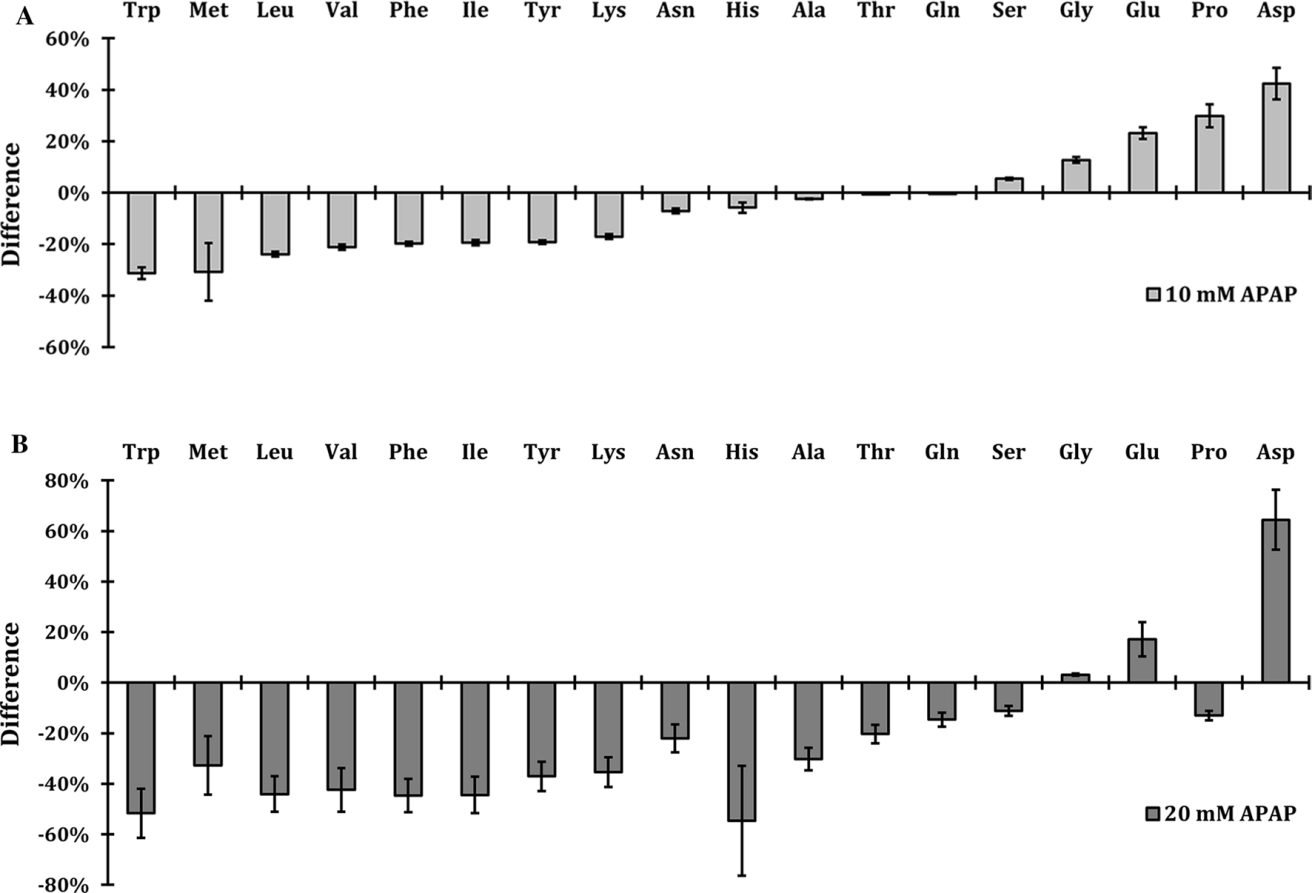
APAP induces changes in amino acid levels in HepG2 cells. The cells were treated with 10 **a** and 20 mM APAP for 2 h **b** and intracellular amino acid levels were measured by HPLC. The bars represent relative decrease/increase compared to the non-treated cells. The error bars represent standard error of measurements of two independent biological samples. The amino acids were ordered based on the highest decrease at 10 mM APAP

Finally, we compared these findings to the human hepatoma cell line HepG2. We cultured HepG2 cells in the presence of 10 and 20 mM APAP and measured the intracellular amino acid levels by HPLC. Interestingly, the results revealed that, similar to yeast, APAP also induced significant changes in amino acid levels in HepG2 cells in a dose-dependent manner (Fig. 8a, b). The highest decrease was measured for Trp, Met, Leu, Val, Phe, Ile, Tyr and Lys (10 mM APAP), with an increase in levels of Gly and Glu (Fig. 8a). The decreased amino acids are all essential in human cells (except for Tyr, which is conditionally essential), suggesting that decrease in amino acid levels probably occurred because of impaired uptake. A striking difference was in the levels of Asp, which were highly reduced in yeast and highly increased in human cells. At the concentration of 20 mM, all amino acid levels were decreased except for Asp and Glu (Fig. 8b). Note that Cys and Arg were under the detection level in these samples. The raw data and the calculations are presented in Online resource 4.

## Discussion

In our previous research, we made a link between ubiquitin levels and APAP toxicity in yeast (Huseinovic et al. 2017b) and showed, using a DUB deletion strain screen (Huseinovic et al. 2017a), that APAP had a comparable toxicity profile as tyrosine and an overlap with drugs causing internalization of high affinity AAP Tat2 (rapamycin and quinine) (Beck et al. 1999; Khozoie et al. 2009). In this study, we explored these observations further by investigating whether APAP can cause a nutrient starvation response in yeast and alter the intracellular concentration of amino acids.

We showed that APAP caused degradation of high affinity AAPs Tat1, Tat2, Mup1 and Hip1, and upregulation of general AAP Gap1: hallmarks of a nutrient starvation response. Because only overexpression of Tat1 and Tat2 conferred resistance to APAP, which are both transporters of aromatic amino acids Trp and Tyr, this points to a specific shortage of (an) aromatic amino acid(s), which cannot be provided by Mup1 and Hip1 with an alternative amino acid specificity (methionine and histide, respectively). Why overexpression of the general permease Gap1 is not able to rescue the growth is unclear. A possible explanation is that Gap1 is only expressed during initial nutrient starvation response, but cannot complement for Trp shortage during prolonged incubations in the presence of APAP. Moreover, Tat2 permease was not degraded in the ubiquitin-deficient and APAP resistant strains *ubp6∆*, *doa4∆* and *ubi4∆* (Fig. 3). Complementation of the medium with individual amino acids demonstrated that Trp levels were indeed crucial for the growth during APAP exposure, while addition of Tyr and Phe enhanced APAP-induced toxicity. Tyr and Phe enhanced toxicity of APAP, probably due to 1) downregulation of Tat1 and Tat2 expression and 2) competitive inhibition of Trp import, both leading to a Trp-deficiency. Accordingly, Trp auxotrophic strain *trp1∆* was hypersensitive to APAP (Fig. 4).

Although overexpression of both Tat1 and Tat2 conferred resistance to APAP, only the presence of Tat1 was crucial for the growth rescue by addition of Trp and *tat1∆* was more sensitive to APAP than *tat2∆* (Fig. 5a, b), which had similar growth properties as WT upon APAP exposure (Fig. 4a–d). Moreover, *tat1∆* strains could only be rescued by addition of Leu, while Trp auxotrophic strain *trp1∆* showed benefit from addition of Trp and Leu. The importance of Leu and Trp might be explained by the substrate preference of Tat1, which transports Leu, Trp, Ile, Val, and Tyr (Schmidt et al. 1994; Regenberg et al. 1999) suggesting that Tat1 is the main transporter of Trp under these conditions. Similar effect is shown for treatment with volatile anesthetic isoflurane in yeast where Tat1 overexpression also conferred resistance and addition of Trp and Leu rescued growth (Palmer et al. 2002). While WT, *trp1∆, tat1∆* and *tat2∆* strains were clearly showing growth impairment upon Tyr and Phe addition during APAP treatment, *tat1∆* also showed impaired growth upon addition of several other amino acids (Met, His, Lys and Thr) suggesting a complex role of Tat1 in regulation of amino acid uptake.

The consequence of APAP exposure and its induced internalization of AAPs were visualized by quantifying the concentrations of amino acids in both yeast and HepG2 cells. We showed a clear change in intracellular amino acid concentrations upon APAP exposure, which was clearly less in ubiquitin-deficient strains *ubi4∆*, *doa4∆* and *doa1∆*, consistent with the unaffected expression levels of Tat2 in these strains. Amino acid levels of Leu, Met, Phe, Tyr, Arg, Asp, Lys, Trp and His in WT cells were already decreased after 10 min of APAP treatment, while concentrations of Gln, Gly and Thr were increased. The high decrease in Leu, Met and His may be due to the fact that WT cells are auxotrophic for these amino acids. However, the addition of these amino acids did not rescue the growth during APAP treatment, except for Leu, which had a slight growth rescue in *trp1*∆ strain (Fig. 4d). Accordingly, overexpression of Mup1 (AAP of Met) and Hip1 (AAP of His) also did not rescue the growth indicating further that the depletion of these amino acids was not a primary cause of the nutrient starvation and that their concentrations did not drop below a critical threshold limiting growth. Although yeast is prototrophic for Trp, Phe and Tyr, we measure a high decrease in these amino acid levels probably because they are the most costly amino acids to synthetize and cells will preferably rely on uptake to obtain sufficient levels. To synthetize 1 mol of Trp, Tyr and Phe, 78, 63 and 56 molecules of ATP are needed, respectively (Barton et al. 2010). These three amino acids are also low abundant, especially Trp, which is 5–10 times less abundant than the next low abundant amino acids (Tyr and Met). This could explain why the levels of Trp are so critical after APAP exposure.

The observed shift to Glu and Gln upon APAP treatment could be explained by the shift from fermentable growth conditions (which yeast undergoes under sufficient nutrient availability) to respiratory growth (which occurs during nutrient starvation). It has been reported that Gly, Ser, Ala, Leu, Val, Tyr, Met, Ile, Thr, and Phe showed the highest concentrations upon fermentable conditions and decreased during a switch to respiratory growth (TCA cycle). Glu reached its highest value under respiratory conditions (Martíez-force and Benítez 1992) and Glu biosynthesis was induced during nutrient starvation response regulated through the retrograde pathway and RTG genes (Johnson et al. 2014). Interestingly, a study with human astrocytes showed that halothane, another volatile anesthetic similar in chemical structure to isoflurane, induced an increase in intracellular Glu (Miyazaki et al. 1997). In our previous study, we showed that deletion of RTG genes RTG1, RTG2, RTG3 and MKS1, conferred resistance to APAP (Huseinovic et al. 2017b).

Finally, we were able to show a similar reduction in amino acid levels in HepG2 cell upon APAP exposure two concentrations: 10 and 20 mM, which are often used to study APAP toxicity in human hepatocytes (Utkarsh et al. 2016; Sison-Young et al. 2017). Unlike primary human hepatocytes, HepG2 cells have a very low P450 activity. For comparison, expression levels of CYP2E1 and CYP3A4 (the enzymes mostly involved in APAP metabolism) have been reported to be ~ 50-fold and ~ 200-fold lower, respectively, in HepG2 cells when compared to human primary hepatocytes (Gerets et al. 2012). We showed a dose-dependent decrease in the intracellular pool of all amino acids except Gly, Glu and Asp. At 10 mM APAP, the highest decrease was measured for Trp, Met, Leu, Val, Phe, Ile, Tyr and Lys. These amino acids are all essential in humans except for Tyr, which is conditionally essential, indicating that it is possibly the uptake inhibition that initially occurs instead of biosynthesis inhibition. In humans, aromatic amino acids Trp, Tyr and Phe are transported by TAT1 transporter expressed in gut endothelial cells and other tissues (Palego et al. 2016). Assuming that the inhibition of Trp by APAP is based on the resemblance of chemical structures of APAP and tyrosine, it is possible that similar inhibition of Trp uptake by APAP could also take place on human aromatic amino acid transporter Tat1. A recently established defective mice (*tat1*^−*/*−^) model (Mariotta et al. 2012) could be used to test its role in APAP toxicity and to see whether addition of different amino acids, especially Trp, Tyr and Phe, would have a similar effect of toxicity as in yeast. Amino acids are precursors of many compounds which are involved in metabolism and neurotransmitters synthesis such as serotonin and melatonin (Trp), dopamine and thyroid hormones (Tyr), Glu which is a neurotransmitter for GABA receptor and many others (Palego et al. 2016). Malnutrition is a high risk for APAP-induced toxicity (Kurtovic and Riordan 2003), which might be linked to reduced nutrient availability upon APAP exposure.

The link between Trp depletion and sensitivity is not only specific for rapamycin (Beck et al. 1999) and APAP, but has been observed for a variety of drugs and conditions such as quinine (Khozoie et al. 2009), ibuprofen (He et al. 2014), immunosuppressors FTY720 (Welsch et al. 2003) and FK560 (Heitman et al. 1993; Schmidt et al. 1994), volatile anesthetics (Palmer et al. 2002) and high pressure (Beck et al. 1999; Palmer et al. 2002; Welsch et al. 2003; Khozoie et al. 2009; He et al. 2014). Inhibition of the TOR pathway (rapamycin) and competitive inhibition of Trp uptake on amino acid transporters (quinine and ibuprofen) have been described as mechanisms that can induce nutrient starvation.

In conclusion, we showed that APAP in yeast leads to loss of high affinity AAPs and has an effect on amino acid availability. The availability of Trp was important for APAP-induced toxicity and non-toxic additions of Tyr and Phe drastically contributed to the toxicity. Moreover, we were able to translate these findings to human cell line HepG2. Therefore, it would be desirable to investigate whether APAP has an effect on nutrient availability in humans, leading to a better understanding of APAP toxicity.

## Electronic supplementary material

Online Resource 1 Raw data and calculations of growth rate measurements of WT, trp1∆, tat1∆ and tat2∆ treated with APAP and supplemented with different amino acids presented in Fig. 4 and 5

Online Resource 2 Raw data and calculations of amino acid levels by HPLC of WT cells treated with APAP for 10, 30 and 60 minutes presented in Fig. 6

Online Resource 3 Raw data and calculations of amino acid levels by HPLC of WT, and ubiquitin-deficient strains ubi4∆, doa4∆ and doa1∆ presented in Fig. 7

Online Resource 4 Raw data and calculations of amino acid levels by HPLC of human hepatoma cells HepG2 treated with 10 and 20 mM APAP presented in Fig. 8

Below is the link to the electronic supplementary material.

Supplementary material 1 (XLSX 292 kb)

Supplementary material 2 (XLSX 48 kb)

Supplementary material 3 (XLSX 45 kb)

Supplementary material 4 (XLSX 23 kb)
